# Private Drinking Water Wells as a Source of Exposure to Perfluorooctanoic Acid (PFOA) in Communities Surrounding a Fluoropolymer Production Facility

**DOI:** 10.1289/ehp.1002503

**Published:** 2010-10-04

**Authors:** Kate Hoffman, Thomas F. Webster, Scott M. Bartell, Marc G. Weisskopf, Tony Fletcher, Verónica M. Vieira

**Affiliations:** 1 Department of Environmental Health, Boston University School of Public Health, Boston, Massachusetts, USA; 2 Program in Public Health, University of California–Irvine, Irvine, California, USA; 3 Department of Environmental Health, Environmental and Occupational Medicine and Epidemiology, Harvard School of Public Health, Boston Massachusetts, USA; 4 London School of Hygiene and Tropical Medicine, London, United Kingdom

**Keywords:** drinking water, perfluorooctanoic acid (PFOA, or C8), pharmacokinetic modeling, private wells, serum

## Abstract

**Background:**

The C8 Health Project was established in 2005 to collect data on perfluorooctanoic acid (PFOA, or C8) and human health in Ohio and West Virginia communities contaminated by a fluoropolymer production facility.

**Objective:**

We assessed PFOA exposure via contaminated drinking water in a subset of C8 Health Project participants who drank water from private wells.

**Methods:**

Participants provided demographic information and residential, occupational, and medical histories. Laboratory analyses were conducted to determine serum-PFOA concentrations. PFOA data were collected from 2001 through 2005 from 62 private drinking water wells. We examined the relationship between drinking water and PFOA levels in serum using robust regression methods. As a comparison with regression models, we used a first-order, single-compartment pharmacokinetic model to estimate the serum:drinking-water concentration ratio at steady state.

**Results:**

The median serum PFOA concentration in 108 study participants who used private wells was 75.7 μg/L, approximately 20 times greater than the levels in the U.S. general population but similar to those of local residents who drank public water. Each 1 μg/L increase in PFOA levels in drinking water was associated with an increase in serum concentrations of 141.5 μg/L (95% confidence interval, 134.9–148.1). The serum:drinking-water concentration ratio for the steady-state pharmacokinetic model was 114.

**Conclusions:**

PFOA-contaminated drinking water is a significant contributor to PFOA levels in serum in the study population. Regression methods and pharmacokinetic modeling produced similar estimates of the relationship.

Perfluorooctanoic acid (PFOA, or C8) is a synthetic chemical that is used as a processing aid in the manufacture of fluoropolymers. Products made with fluoropolymers possess unique properties, including oil, stain, grease, and water repellency. These properties led to the widespread use of fluoropolymers in a number of products, including nonstick cookware, weather- and stain-resistant clothing and textiles, building and construction materials, and electronics (Renner 2001).

The chemical structure of PFOA makes the compound extremely resistant to environmental and metabolic degradation. PFOA has been detected globally in the environment (Lau et al. 2007). It is well established that PFOA is readily absorbed via inhalation and ingestion. Routes of exposure in the general population remain unclear, although research suggests that diet is a potentially important source (Trudel et al. 2008). PFOA is detected in the vast majority of serum samples from U.S. and world populations (Lau et al. 2007). Once absorbed, PFOA is eliminated from the human body very slowly. Estimates of the serum half-life of PFOA range from 2.3 years in residents of a contaminated community to 3.8 years in retired fluorochemical workers (Bartell et al. 2010; Olsen et al. 2007). Some evidence suggests that PFOA concentrations in serum are declining, possibly due to reductions in use; however, the median serum concentrations remain around 4 μg/L in the U.S. population (Calafat et al. 2007a, 2007b; Olsen et al. 2007). PFOA exposure also has been linked to a variety of health impacts in animals, including increased cancer risk, adverse reproductive outcomes, and liver damage (Lau et al. 2004, 2007). Because of a lack of data, health impacts of exposure in humans remain largely unknown (Steenland et al. 2010).

In 2003, the U.S. Environmental Protection Agency (EPA) began an enforceable consent agreement process with industry and other stakeholders to collect additional information for a PFOA risk assessment (U.S. EPA 2010a). The U.S. EPA and DuPont (the maker of Teflon) entered a memorandum of understanding (MOU) in November 2005 as part of the risk assessment. Building on an agreement in place between the West Virginia Department of Environmental Protection and DuPont, the MOU required DuPont to conduct environmental sampling, including the monitoring of groundwater and surface waters around its Washington Works facility in Parkersburg, West Virginia, USA (U.S. EPA 2004a).

DuPont began using PFOA in the manufacture of Teflon at its Washington Works plant in the early 1950s. According to data provided by the company, emissions to air and the Ohio River reached a maximum in the late 1990s (Emmett et al. 2006; Paustenbach et al. 2007). The company reported a large reduction in these emissions in recent years (U.S. EPA 2010b). Previous research indicates that the primary source of exposure for individuals in the surrounding communities is contaminated groundwater that is used for drinking water (Emmett et al. 2006; Steenland et al. 2009). The groundwater in the area was contaminated via two main routes: PFOA released into the atmosphere was deposited onto soils and eventually leached downward into groundwater, and PFOA was released directly into the Ohio River, which runs near the facility and is linked to the groundwater supply (Paustenbach et al. 2007).

In 2001, a group of residents in communities surrounding the facility filed a class action lawsuit against DuPont alleging health damages after PFOA was detected in public drinking water. The settlement established the C8 Health Project, a baseline survey conducted in 2005–2006 to investigate potential links between PFOA and human disease in the area surrounding the facility (Frisbee et al. 2009).

Previous studies showed a significant association between living in an area with contaminated public drinking water and increased PFOA levels in serum using water-district–level data (Emmett et al. 2006; Hölzer et al. 2008; Steenland et al. 2009; Vieira et al. 2008. These studies are partially ecologic because the exposure variable is assigned at the group level, whereas other variables are assigned at the individual level (Björk and Strömberg 2002; Webster 2000, 2002). In particular, previous studies provide information on serum concentration in relation to average exposure for populations serviced by the same water supply, but investigations into the relationship between contaminated private household well water and serum levels are lacking. In the present analyses, we examined the relationship between PFOA cocncentrations in serum and in drinking water using data collected from private drinking water wells contaminated by industrial emissions. By using data from private drinking water wells, we were able to quantify PFOA levels in the drinking water of C8 Health Project participants at the individual level. We assessed the relationship using standard regression approaches; for comparison, we also used a pharmacokinetic model to explore the association between PFOA in drinking water and in serum levels. Simple, single-compartment, first-order models have been applied previously to estimate the serum concentration after exposure from diet and drinking water (Fromme et al. 2007; Vieira et al. 2008). In the present analyses, we used updated estimates of pharmacokinetic parameters to predict the serum:drinking-water concentration ratio. We compared the association between drinking-water and serum PFOA concentrations from regression models with those obtained in pharmacokinetic analyses.

## Materials and Methods

### Study population

**Figure 1:**
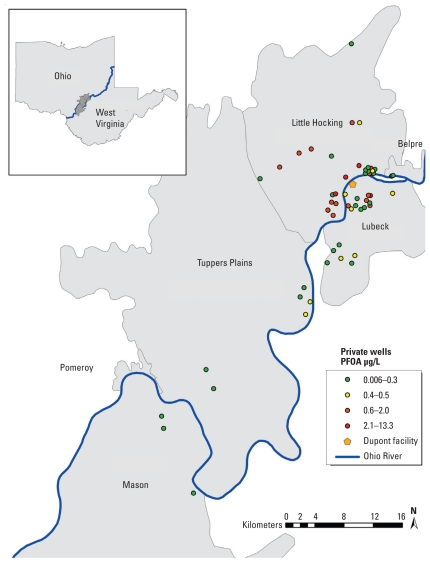
Water districts included in the C8 Health Project and the locations of private drinking water wells that show the average PFOA concentration for each well.

The C8 Health Project, a cross-sectional study of approximately 69,000 adults, was conducted by Brookmar Inc. from August 2005 through August 2006 (Frisbee et al. 2009). Participants lived in one of six public water districts in West Virginia and Ohio that surround DuPont’s Washington Works facility: Belpre, Little Hocking, Lubeck, Mason County, Pomeroy, and Tuppers Plains–Chester (Figure 1). Data were collected from each participant using questionnaires and clinical examinations to obtain demographic information and residential, occupational, and medical histories (Frisbee et al. 2009). Concentrations of 10 perfluorinated compounds, including PFOA, were also determined in serum samples taken once from each participant between August 2005 and August 2006. Detailed analytic methods were described previously (Kuklenyik et al. 2004). Briefly, serum samples were analyzed using automated solid-phase extraction coupled to reversed-phase high-performance liquid chromatography.

The Institutional Review Board of Boston University Medical Center approved this research, and the participants provided informed consent to have their data used for research purposes.

Water monitoring was conducted by DuPont in public and private wells surrounding the Washington Works facility beginning in 2001. Private well monitoring reports contained PFOA measurements as well as the primary use of each well and the name and address of each well’s owner. These reports are available through the EPA (U.S. EPA 2004b). We linked well monitoring data for 62 private wells that were used primarily for drinking water to C8 Health Project participants based on name and address. We also identified individuals who had the same last name and address as the well owner as family members. A total of 115 participants were included in the study. The number of samples taken in each well before the collection of serum samples varied. Although most wells were sampled just once, 11 of the 62 private wells were sampled multiple times.

### Statistical analysis

In preliminary analyses, we identified several participants with serum-PFOA concentrations or well-PFOA concentrations that were much greater than those of the other participants. For data with outliers, using standard least squares estimation is both inefficient and biased; regression coefficients are pulled toward outliers, and estimates of the variance are artificially inflated, which can obscure outliers (Hampel et al. 1986). Therefore, we used robust regression methods to assess the relationship between PFOA concentrations in serum and in drinking water. Robust regression provides stable results by limiting the influence of outliers and is generally less subject to bias than are standard least squares estimation methods (Hampel et al. 1986). Robust regressions were performed using Yohia’s MM estimator, which possesses high statistical efficiency and provides stable estimates of regression parameters when data include a relatively large percentage of outliers (Yohai 1987).

Additionally, because multiple individuals from the same family were included in the analyses, which violates the assumption of independence for linear regression, we used generalized estimating equations (GEEs) in a second set of analyses to predict PFOA concentrations in serum from PFOA concentrations in drinking water. Using GEEs, we account for possible residual within-family correlation and investigate the sensitivity of our results from the robust regression that includes multiple individuals from the same family in the analyses. GEEs and robust regressions were preformed using SAS version 9.1 (SAS Institute Inc., Cary, NC, USA).

Age and sex have been previously associated with serum PFOA levels in the population surrounding the Washington Works facility, as well as in other populations (Emmett et al. 2006; Hölzer et al. 2008; Steenland et al. 2009). Additionally, working at the Washington Works plant and growing one’s own vegetables were linked to increased PFOA levels in serum (Emmett et al. 2006; Steenland et al. 2009). We included these a priori variables in all statistical models. We also assessed a number of other variables that have been linked to serum PFOA levels: body weight, consuming bottled water (modeled as yes or no), smoking cigarettes, and drinking alcohol (Emmett et al. 2006; Steenland et al. 2009). Only the a priori variables were included in the final models because the others did not materially alter the association between serum and well-PFOA levels (did not cause a change > 10% in the predicted contribution of drinking water to serum).

For wells with multiple PFOA sampling events, we used the arithmetic average PFOA concentration in each well to predict serum levels in regression models. This method provided an estimate of the serum:drinking-water concentration ratio that is readily comparable to the results of a steady-state pharmacokinetic model that assumes that the concentration of PFOA in drinking water is constant over time (discussed in the next section). We also performed an analysis using time-weighted water concentrations based on a non-steady-state pharmacokinetic model. In the main analyses, we included all individuals regardless of how long they had lived at their current residence. We also performed analyses investigating the sensitivity of our results to the residential duration at a particular well. By restricting the sample to long-term residents (> 15 years), we ensured that participants had been exposed to water from a specific well long enough for their serum levels to have reached steady state.

### Pharmacokinetic models

Regression, after adjusting for other factors, provides us with an estimate of the change in serum concentrations per unit change in water concentration. For comparison with the regression analyses, we also predicted the ratio of serum to PFOA concentrations in drinking water using a simple first-order, single-compartment pharmacokinetic model. Bartell et al. (2010) previously demonstrated that the pharmacokinetics of PFOA in humans is consistent with first-order elimination. Based on data that suggested that the duration of exposure to PFOA-contaminated drinking water in the study population is on the order of decades (Paustenbach et al. 2007), we assumed that levels of PFOA in serum had reached a steady-state concentration. The ratio of steady-state serum PFOA concentration, *C_s_* (micrograms per liter), to water concentration, *C_w_* (micrograms per liter), was modeled using the following equation (Bartell 2003):


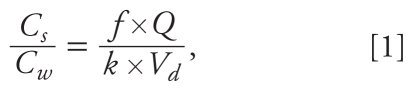


where *f* is the fraction of PFOA absorbed, *Q* is the daily water intake (liters per day), *k* is the first-order rate constant for PFOA elimination (day^−1^; *k* = 0.693/*t*_1/2_, where *t*_1/2_ is the half-life), and *V_d_* is the apparent volume of distribution (liters).

Values for each parameter were obtained from a review of available animal and human PFOA pharmacokinetic data (Table 1). We assumed that 100% of ingested PFOA was absorbed based on animal data (Butenhoff et al. 2004; Gibson and Johnson 1979; Hundley et al. 2006). Similar estimates of the fraction of PFOA absorbed in humans that are highly exposed to PFOA have been used previously (Thompson et al. 2010; Trudel et al. 2008). In previous pharmacokinetic analyses of PFOA (Vieira et al. 2008), we used a serum half-life of 3.8 years (1,388 days) based on a small study of retired fluorochemical production workers (Olsen et al. 2007). In the present analyses, we applied a more recent estimate of 2.3 years (840 days) based on data from Bartell et al. (2010) collected in a subset of C8 Health Project participants. The volume of distribution (*V_d_*) is a proportionality constant in pharmacokinetic modeling that relates the total amount of a chemical in the body to the concentration in plasma. We used a *V_d_* for PFOA of 181 mL/kg and 198 mL/kg for males and females, respectively, based on results from cynomolgus monkey experiments (Butenhoff et al. 2004). Thompson et al. (2010) proposed a similar *V_d_* (170 mL/kg) using data from residents of two chronically exposed communities around DuPont’s Washington Works facility. Because the goal of our regression analysis was to use the serum and water data to estimate a steady-state ratio, and not the *V_d_*, we used the Butenhoff estimate from monkeys in the pharmacokinetic model rather than the Thompson estimate from the same community. We scaled the *V_d_* to the sex and body weight of the study participants and used the median of the study population in pharmacokinetic models. Because water consumption data were unavailable, we used the U.S. EPA’s recommended average tap water intake rate for adults of 1.4 L/day, which includes water consumed from the tap as a beverage or used in the preparation of foods and beverages (U.S. EPA 1997).

## Results

Linking well-monitoring data to C8 Health Project participants, we were able to identify 115 individuals who used 62 different private wells for drinking water. Of these individuals, 4 (3.5%) were missing data (PFOA levels in serum: *n* = 1, body weight: *n* = 2, race/ethnicity: *n* = 1) and were excluded from the analyses. We also excluded vegetarians (*n* = 2) and nonwhite participants (*n* = 1) because the numbers were too small to adequately control for these variables. Our final sample consisted of 108 participants. Serum PFOA levels ranged from 0.9 to 4751.5 μg/L, with a median concentration of 75.7 μg/L (mean ± SD, 177.3 ± 499.7 μg/L). As reported previously in the larger C8 Health Project sample (Steenland et al. 2009), individuals who grew their own vegetables and who were employed at DuPont had higher median serum PFOA concentrations (Table 2). PFOA concentrations were higher among older (> 65 years) and heavier (> 80 kg) participants, but the differences were not statistically significant (Table 2).

Figure 1 shows the well locations and the corresponding average PFOA concentrations. The number of participants using each well ranged from 1 to 4. The median PFOA concentration in drinking water wells included in our analyses was 0.2 μg/L (mean ± SD, 0.8 ± 1.9 μg/L). Although the median was below the U.S. EPA provisional health advisory level of 0.4 μg/L, many participants had drinking water levels that exceeded the advisory level (U.S. EPA 2009). We found considerable variability between wells, with PFOA concentrations ranging from below the limit of quantification (LOQ = 0.006 μg/L) farthest from the Washington Works facility to 13.3 μg/L closest to the facility. One sample was reported below the LOQ and was assigned the LOQ (0.006 μg/L) in the analyses. Multiple samples were taken from 11 wells that were used by 19 study participants. In general, we did not find an overall trend from 2001 to 2005 in the concentrations of PFOA in private drinking water. Although PFOA concentrations in each well appeared to fluctuate by season, these differences may be due to seasonal changes in precipitation. PFOA concentrations measured in 2004 and 2005 for a subset of wells measured seasonally are shown in Supplemental Material, Figure 1 (doi:10.1289/ehp.1002503).

### Regression results

We examined the shape of the relationship between serum-PFOA concentration and average drinking water PFOA concentration using a locally weighted regression smoother (LOESS) in S-Plus (version 8.0; Tibco Software, Inc., Palo Alto, CA, USA). Visual inspection of a plot of the smoothed data indicated that the association between serum and drinking water PFOA levels could be estimated as a linear trend (data not shown), as suggested by the pharmacokinetic model (Equation 1). We therefore included the average drinking water PFOA concentration as a linear predictor of nontransformed serum PFOA concentrations in regression models. In the adjusted robust regression models each micrograms per liter increase in drinking water PFOA concentration was associated with a 141.5 μg/L [95% confidence interval (CI) = 134.9–148.1] increase in serum concentrations. Table 3 presents effect estimates for other variables included in the model. Growing one’s own vegetables, being male, and being employed at DuPont were associated with elevated serum PFOA levels; however, associations did not reach statistical significance at the 0.05 level. The estimated background serum level in this population after accounting for known sources was 7.4 μg/L (Table 3). Additionally, we investigated differences in the serum:drinking-water concentration ratio in males and females. When we stratified by sex, we observed very similar ratios in both sexes. Accordingly, we did not observe a significant (*p*-value < 0.05) sex-by-water concentration interaction (data not shown) when we included an interaction term in the models.

Robust regression analyses revealed six outliers (observations for which the standardized residual was > 3). For these individuals, the predicted values for serum PFOA concentrations using regression parameters underestimated or overestimated observed concentrations (standardized residuals, 3.0–44.5). In the analyses using GEEs, we observed a small within-family correlation of serum PFOA levels of 0.1. Compared with the results of the robust regression, GEE analyses that excluded outliers produced a very similar estimate of effect (β) for each 1 μg/L increase in PFOA concentration (β = 141.8 μg/L; 95% CI, 134.3–149.4 μg/L) in drinking water. When we included outliers in the GEE, the estimate of the association between PFOA levels in serum and in drinking water was much larger. The inclusion of one participant in particular, with the highest PFOA concentrations in serum and drinking water in the population, increased the estimate of effect to 232.7 μg/L (95% CI, 200.9–264.5 μg/L). We could not identify a plausible explanation for this participant’s extreme serum concentration using available data (the participant did not report being employed in the fluorochemical industry). Increased water consumption in this individual may have resulted in the extreme concentration; however, data were not available to evaluate this hypothesis.

When we restricted our analyses to individuals with a residency > than 15 years, our results were similar (β = 140.2 μg/L; 95% CI, 132.1–148.4 μg/L; *n* = 67). We considered other residential duration restrictions (2, 5, 10, and 20 years), but restrictions had little effect on the magnitude of the association between serum and drinking water. We also excluded participants who were ever employed at the Washington Works facility, because these individuals may have had other significant sources of exposure. Again, the association between drinking water levels and serum was similar when we excluded these individuals. Additionally, excluding participants who reported consuming bottled water (*n* = 6) from analyses had little effect on the magnitude of the association between serum and drinking water.

### Comparison of pharmacokinetic and regression results

Using the simple steady-state first-order pharmacokinetic model (Equation 1) with a median *V_d_* of 15,000 mL in the study population after scaling for the body weight and sex of study participants, we obtained a serum:drinking-water concentration ratio of 114. This ratio is similar to the ratio of 141.5 that was derived from regressing serum concentrations versus water concentrations.

## Discussion

Serum PFOA concentrations in users of private wells in the area surrounding DuPont’s Washington Works facility were much greater than those observed in the general U.S. population and were comparable to what has been observed in the study area previously (Emmett et al. 2006; Steenland et al. 2009; Vieira et al. 2008). Private drinking water wells in the area were contaminated with PFOA, with levels in some wells being much greater than those observed in public drinking water supplies in the same area, which ranged from 0.03 μg/L in Mason County to 3.5 μg/L in Little Hocking (Emmett et al. 2006; Steenland et al. 2009). Using data from private wells, we had a large number of individual exposure levels and were able to assess a wide range of exposures to PFOA via drinking water.

The results of the regression analyses are consistent with a strong association between PFOA levels in serum and PFOA concentrations in drinking water. We found little difference in the association between serum and drinking water PFOA concentrations when we limited our analyses to 67 individuals who were long-term residents.

The serum:drinking-water concentration ratio of 141.5, which was estimated using regression analysis, was similar to ratios obtained in previous partially ecologic analyses (Emmett et al. 2006; Vieira V, Webster T, Bartell S, Steenland K, Savitz D, Fletcher T, unpublished data). In our previous work in the study area, we found serum:drinking-water concentration ratios in public water districts ranging from 59 to 411 (Vieira V, Webster T, Bartell S, Steenland K, Savitz D, Fletcher T, unpublished data). In Little Hocking, Ohio, near the Washington Works facility, Emmett et al. (2006) estimated a water concentration ratio of 105 in an analysis of public water consumers. Additionally, in a small sample of private well users (*n* = 6), serum:water concentration ratios ranged from 142 to 855 (Emmett et al. 2006).

The steady-state serum:drinking-water concentration ratio of 114 obtained from pharmacokinetic modeling was close to the estimate of effect (141.5) obtained from regression analyses. This result suggests that the pharmacokinetic model provides a reasonable estimate. We used a serum PFOA half-life based on data that Bartell et al. (2010) collected in a subset of C8 Health Project participants with exposure levels and patterns similar to the participants in our analyses. Using the half-life estimate from Olsen et al. (2007) of 3.8 years increased the serum:drinking-water ratio to 188. Other pharmacokinetic parameters that we used were more uncertain, particularly the volume of distribution, which we estimated based on animal data (Butenhoff et al. 2004). A recent study by Thompson et al. (2010) estimated a very similar *V_d_* (170 mL/kg) based on data from community residents; using the *V_d_* from that study produced a similar serum:drinking-water concentration ratio of 126. Based on data from subchronic toxicity studies in monkeys, however, Washburn et al. (2005) recommended using a volume of distribution that was a factor of 10 higher than the Butenhoff et al. (2004) ratio that we used in our analyses. If we had used their volume of distribution, our ratio would have been reduced by an order of magnitude. Further research is needed on the volume of distribution of polyfluoroalkyl chemicals in humans. Additionally, in the absence of consumption data for each individual, we used the U.S. EPA estimated average daily tap water consumption value of 1.4 L/day; however, water consumption in the study population likely varied (U.S. EPA 1997). We believe that the difference in serum:drinking-water concentration ratio estimates from regression and pharmacokinetic models may be explained by these uncertainties.

As reported previously for C8 Health Project participants, we observed a positive association between serum PFOA levels and growing one’s own vegetables after adjusting for water concentration, suggesting that consuming locally grown food may be an important source of exposure in this population (Bartell et al. 2010; Steenland et al. 2009). The background serum PFOA concentration predicted in regression analyses (7.4 μg/L) is greater than background levels previously reported in the U.S. population [geometric mean, 3.8 μg/L (Calafat et al. 2007b); arithmetic mean, 4.3 μg/L (Centers for Disease Control and Prevention 2007)]. These results suggest that there may be other sources of PFOA exposure in the C8 Health Project population that we did not include in the model or that random exposure misclassification may be inflating the predicted background levels for this population. Other potentially important sources of PFOA exposure in this population include water consumption at work, school, or religious and social organizations frequented by study participants. Although the release of PFOA from the Washington Works facility has been reduced (U.S. EPA 2010b), PFOA may still be present in indoor environments and may contribute an additional source of exposure for residents. Data were not available to test hypotheses on these exposure sources.

Our analyses are limited by our steady-state assumption and reliance on a single measurement of serum levels and, in most cases, a single measurement of drinking water PFOA levels. For a small number of individuals with multiple well measurements, we considered variability in well measurements in a sensitivity analysis using a time-weighted well concentration rather than an arithmetic average to predict serum PFOA concentrations [see Supplemental Material (doi:10.1289/ehp.1002503)]. Although we found some seasonal variability from 2001 to 2005, on average PFOA concentrations in the wells were fairly stable, and we found no long-term trend during this time period. Consequently, predicted serum concentrations that accounted for variation in PFOA concentrations in wells were similar to those obtained using simple steady-state models (data not shown).

Despite these limitations, our analyses have a number of strengths. We were able to link PFOA measurements in drinking water to a relatively large number of individual study participants who consumed private well water. The extensive questionnaire (Frisbee et al. 2009) administered as part of the C8 Health Project allowed us to consider a number of potential confounders in the association between PFOA levels in serum and in drinking water (including age, sex, growing one’s own vegetables, body weight, bottled water consumption, cigarette smoking, and alcohol consumption). Unlike previous assessments, which used water samples from public water supplies, we used drinking water samples from the participants’ wells, which increased the variability of exposure measures. Additionally, available residential history information allowed us to consider differences in long and short-term residents using contaminated wells for drinking water.

## Conclusions

Private drinking water wells in West Virginia and Ohio communities surrounding the DuPont Washington Works facility are contaminated with PFOA. Concentrations in private wells are, in some cases, much greater than those observed in area public water districts. For private well users, adjusted regression analyses indicate that PFOA levels in drinking water are a significant predictor of PFOA levels in serum. The regression analysis predicted a 141.5 μg/L increase in serum levels for each 1 μg/L increase in drinking water PFOA—a very similar result to the 114 μg/L in serum for each 1 μg/L predicted in steady-state pharmacokinetic models. These results may also be applicable in other areas with point-source PFOA contamination.

## Correction

In the manuscript originally published online (second paragraph of “Results”), the U.S. EPA provisional health advisory level for PFOA was given as 0.04 μg/L; however, this level is actually 0.4 μg/L. Thus, the median PFOA concentration found in the present study was “below” instead of “much greater than” the U.S. EPA provisional health advisory level. It has been corrected here.

## Figures and Tables

**Table 1:** Pharmacokinetic parameter values and sources.

Parameter	Symbol	Value	Data source
Water intake	*Q*	1.4 L/day	U.S. EPA 1997
Fraction of PFOA absorbed^a^	*f*	100%	Gibson and Johnson 1979
Half-life	*t*_1/2_	2.3 years, 840 days	Bartell et al. 2010
Volume of distribution^a^	*V_d_*	Male, 181 mL/kg; Female, 198 mL/kg; multiplied by individual body weight	Butenhoff et al. 2004

a Based on animal data.

**Table 2:** Selected population characteristics, serum PFOA concentrations, and statistical significance of difference.

Characteristic	*n* (%)	Median serum PFOA [μg/L (interquartile range)]	*p*-Value
Total population	108 (100)	75.7 (31.5–130.5)	
Male	51 (47.2)	82.2 (45.9–164.3)	0.10
Female	57 (52.8)	68.1 (21.0–115.5)	
Grow own vegetables			
No	64 (59.3)	50.7 (24.9–107.3)	< 0.001
Yes	44 (40.7)	91.2 (57.0–145.2)	
Employed at DuPont			
No	94 (87.0)	67.6 (72.2–102.4)	0.11
Yes	14 (13.0)	87.1 (27.4–145.1)	
Age (years)			
≤ 65	63 (58.3)	59.8 (20.6–115.9)	0.35
> 65	45 (42.7)	84.9 (49.0–145.1)	
Body weight (kg)			
≤ 80	50 (46.3)	63.5 (31.5–107.7)	0.64
> 80	58 (53.7)	81.2 (30.1–177.4)	

**Table 3:** Adjusted^a^ robust regression model of serum PFOA.

Covariate	β-Coefficient (95% CI)
Intercept	7.4 (−9.8 to 24.4)
Well PFOA	141.5 (134.9 to 148.1)
Males	18.8 (−1.6 to 39.1)
Age > 65 years	−4.2 (−24.2 to 15.9)
Grow own vegetables	18.4 (−1.3 to 38.1)
Employed at DuPont	5.9 (−24.1 to 36.2)

a The inclusion of other covariates (body weight, bottled water consumption, cigarette smoking, and alcohol consumption) did not alter the main associations.
